# The inflammatory response and neuronal injury in *Streptococcus suis* meningitis

**DOI:** 10.1186/s12879-018-3206-6

**Published:** 2018-07-03

**Authors:** Jana Seele, Simone C. Tauber, Stephanie Bunkowski, Christoph G. Baums, Peter Valentin-Weigand, Nicole de Buhr, Andreas Beineke, Asparouh I. Iliev, Wolfgang Brück, Roland Nau

**Affiliations:** 1 Department of Neuropathology, University Medical Center Göttingen, Georg-August-University Göttingen, Göttingen, Germany; 2 Department of Geriatrics, Evangelisches Krankenhaus Göttingen-Weende, Göttingen, Germany; 3 Department of Neurology, RWTH University Hospital, Aachen, Germany; 4 Institute for Bacteriology and Mycology, Center for Infectious Diseases, Faculty of Veterinary Medicine, University Leipzig, Leipzig, Germany; 5 Institute for Microbiology, Center for Infection Medicine, University of Veterinary Medicine Hannover, Hannover, Germany; 6 Department of Physiological Chemistry, Department of Infectious Diseases, University of Veterinary Medicine Hannover, Hannover, Germany; 7 Research Center for Emerging Infections and Zoonoses (RIZ), University of Veterinary Medicine Hannover, Hannover, Germany; 8 Department of Pathology, University of Veterinary Medicine Hannover, Hannover, Germany; 9 Institute of Anatomy, University of Bern, Bern, Switzerland

**Keywords:** *Streptococcus suis*, Pig model, Meningitis, Intranasal infection, Immunohistochemistry

## Abstract

**Background:**

Many of the currently used models of bacterial meningitis have limitations due to direct inoculation of pathogens into the cerebrospinal fluid or brain and a relatively insensitive assessment of long-term sequelae. The present study evaluates the utility of a *Streptococcus* (*S*.) *suis* intranasal infection model for the investigation of experimental therapies in meningitis.

**Methods:**

We examined the brains of 10 piglets with *S. suis* meningitis as well as 14 control piglets by histology, immunohistochemistry and in-situ tailing for morphological alterations in the hippocampal dentate gyrus and microglial activation in the neocortex.

**Results:**

In piglets with meningitis, the density of apoptotic neurons was significantly higher than in control piglets. Moreover, scoring of microglial morphology revealed a significant activation of these cells during meningitis. The slight increase in the density of dividing cells, young neurons and microglia observed in piglets suffering from meningitis was not statistically significant, probably because of the short time frame between onset of clinical signs and organ sampling.

**Conclusions:**

The morphological changes found during *S. suis* meningitis are in accordance with abnormalities in other animal models and human autopsy cases. Therefore, the pig should be considered as a model for evaluating effects of experimental therapeutic approaches on neurological function in bacterial meningitis.

## Background

*Streptococcus* (*S.*) *suis* is an important pathogen in veterinary medicine. Prevention and treatment of *S. suis* infections contribute substantially to the high rate of antibiotic consumption in the pig producing industry [1]. More than 50% of the cases in pigs occur below the age of 12 weeks [2]. *S. suis* can also affect humans. Transmission to humans usually occurs by close contact with sick or asymptomatic carrier pigs or contaminated food. Most human infections are sporadic, but outbreaks took place particularly in Southeast Asia. *S. suis* is the most frequent cause of human bacterial meningitis in pork-consuming and pig-breeding countries in this area [3]. Both in pigs and humans, *S. suis* can cause meningitis, septicemia, endocarditis, arthritis, and septic shock, with a high mortality. Meningitis and septicemia are the most frequent clinical disease entities in humans [4]. The fatality rate in adult humans is approximately 13%, and the most frequent long-term sequelae are hearing loss (39%) and vestibular dysfunction (23%) [5].

Many animal models used to study the pathophysiology of bacterial meningitis and to assess the effect of experimental therapeutic approaches on outcome have two main shortcomings: 1. Bacteria are often injected directly into the cerebrospinal fluid (CSF) or brain [6–9], because with few exceptions [10], intranasal or intravenous modes of administration of bacteria do not cause meningitis in rates high enough to be suitable for experimental studies. 2. The neuropsychological assessment of long-term sequelae, particularly in mice, but also in rats, is not very sensitive to detect deficits. *S. suis* meningitis in pigs as a model may overcome these shortcomings, since infection usually is induced by intravenous injection or intranasal inoculation of *S. suis* which closely mimics the conditions in human disease [11]. From the bloodstream, these pathogens most likely enter the CSF via the choroid plexus [12]. In terms of size, the pig brain corresponds better to humans compared to the rodent brain. Moreover, genetics, anatomy and physiology of the pig are closely related to humans [13]. For the assessment of long-term motor and neuropsychologic sequelae in pigs, several sensitive and valid test batteries are available [14, 15]. For these reasons, the pig model of *S. suis* meningitis may be preferable to all rodent models to study the effect of experimental therapeutic approaches on neurological function in meningitis.

Morphological abnormalities in human autopsy cases and radiological studies as well as animal models of bacterial meningitis are well characterized [7, 8, 16–23]. The present study further evaluates the utility of the piglet model for the study of experimental therapies in bacterial meningitis. We aimed at assessing whether the morphological alterations seen in the brains of piglets with *S. suis* meningitis are comparable to the abnormalities observed in human autopsy cases and in rodent models of bacterial meningitis.

## Methods

### *S. suis* infection model

In this study, brains of piglets from partially published experiments were analysed [24, 25]. Briefly, 35 German Landrace piglets purchased from one breeder at ages of 4 to 9 weeks, free of *suilysin (sly)+, muramidase-released protein* (*mrp*)+, *extracellular factor* (*epf*) + and *capsular serotype* (*cps)*2+ *S. suis* strains, were intranasally infected with approximately 1 × 10^9^ colony forming units (CFU) *S. suis* serotype 2 strain 10 *(sly+, mrp+, epf+)* after decreasing the colonization resistance of the nasal mucosa by intranasal treatment with 1% acetic acid [26]. Piglets were cared for in accordance with the principles outlined in the European Convention for the Protection of Vertebrate Animals Used for Experimental and Other Scientific Purposes. The animal experiments were approved by the Committee on Animal Experiments of the Lower Saxony State Office for Consumer Protection, Food Safety and Animal Protection (permit no. 33.14–42,502–04-12/0965 and 33.9–42,502–04-12/0965). In the case of high fever (≥ 40.5 °C), apathy and anorexia persisting over 36 hours (h) as well as in all cases with clinical signs of acute polyarthritis or severe meningitis, animals were anaesthetized with 2 mg/kg azaperon (Stresnil; Janssen, Neuss, Germany) and 10 mg/kg ketamine-hydrochloride (Ursotamin; Serumwerk, Bernburg, Germany) intramuscularly. Thereafter, they were sacrificed by injection of 100 mg/kg pentobarbital sodium (Release; Wirtschaftsgenossenschaft deutscher Tierärzte eG, Garbsen, Germany) into the marginal ear vein. The interval between infection and termination of the experiment for animals with meningitis is depicted in Table 1.

**Table 1:** Clinical data of piglets with meningitis

animal no.	lesion^a^	intensity of the lesion	semiquantitative bacterial load^b^ in CSF	semiquantitative bacterial load^b^ in brain swabs	age at time point of infection (in weeks)	interval between infection and first signs of sickness (days)^c^	interval between infection and termination of the experiment (days)
1	A	mild	+++	+++	4	1	1.5
2	B; D	severe	+++	+	4	1.5	3
3	B; D	severe	+++	++	4	4	5.5
4	B	severe	+++	+	7	2.5	3.5
5	B	severe	–	+++	9	1	2.5
6	A	mild	++	++	9	1	3
7	B	severe	++	+	9	6.5	7.5
8	C	severe	+++	+++	9	2.5	3.5
9	B	severe	+++	+	9	6.5	7.5
10	B	severe	–	+++	9	1	2.5

^a^A = focal purulent meningitis

B = diffuse purulent meningitis

C = multi-focal purulent meningitis

D = encephalitic involvement of the brain tissue

^b^bacterial load: + < 50 colonies; ++ ≥50 colonies; +++ ≥500 colonies per plate; − no detection of the infection strain

^c^first signs of sickness such as fever (≥ 40.2 °C), apathy, convulsions, lameness and anorexia

Out of 35 infected piglets, 10 animals developed meningitis and were classified as experimental group. A total of 14 piglets that showed no inflammation in the CNS and no gross morphological abnormalities in all organs investigated, or had only histopathological alterations in one organ other than the brain, and in that organ, *S. suis* was not detected, were included in the control group. Animals were excluded from the analysis, as they showed histopathological alterations in more than one organ and/or bacteria were detected in the respective organ(s). These piglets had pleuritis, peritonitis, synovialitis, splenitis, hepatitis, pneumonia or endocarditis. The 14 control piglets were screened negative for *S. suis* serotype 2 strain 10 (with exception of the tonsils). All surviving piglets including the control piglets were killed 14–15 days post infection (dpi). Thereafter, each animal was autopsied according to the same protocol, which included predefined collection of samples for histological and bacteriological investigations. Purulent inflammations were scored by blinded investigators as described previously [26]. The following lesions were detected in the piglets by histological investigations (*n* = 35; some piglets had more than one histopathological lesion): meningitis (*n* = 10, 28.6%; of which 2 piglets showed also encephalitic involvement of the brain tissue), pleuritis or peritonitis (*n* = 7, 20.0%), synovialitis (*n* = 5, 14.3%), splenitis or hepatitis (*n* = 8, 22.9%), pneumonia (*n* = 15, 42.9%) and endocarditis (*n* = 1, 2.9%). Eight piglets (22.9%) did not develop histopathological lesions (with exception of a minimal accumulation of neutrophils in the red pulp of the spleen). For assessment of the semiquantitative bacterial loads 50 μl CSF and the brain swab of each animal were plated on Columbia sheep blood agar and incubated for 24 h at 37 °C (+ < 50 colonies; ++ ≥50 colonies; +++ ≥500 colonies per plate).

Brain sections of the hippocampal formation, the frontal cortex and the cerebellum of 10 piglets with meningitis confirmed by histopathological analysis and 14 control piglets from the same infection experiments were used for further analysis by histology, immunohistochemistry and in-situ tailing. Inflammation of meninges and brain tissue was evaluated by haematoxylin and eosin (HE) and chloroacetate esterase (CAE) staining of 2 μm sections from the frontal cortex, the cerebellum and the hippocampal formation. HE stained sections were also used to detect ischemic lesions.

### Immunohistochemistry and in-situ tailing

Two μm thick brain sections of the hippocampal formation were deparaffinised, microwaved (5 × 3 min; 800 W), incubated in citric acid buffer (10 mmol/L, pH 6.0) for 10 min and then blocked with 10% fetal calf serum (FCS) in phosphate-buffered saline (PBS) for 30 min. All primary antibodies were applied at the concentrations indicated below and incubated overnight at 4 °C in PBS. Brain slices were stained for the detection of proliferating cells with a monoclonal mouse anti-PCNA (proliferating cell nuclear antigen) antibody (dilution 1:200; Chemicon, Temecula, CA), microglia were detected using a polyclonal rabbit anti-Iba-1 (ionized calcium binding adaptor molecule 1) (1:400, Wako, Neuss, Germany) antibody. Astrocytes were visualized by a polyclonal rabbit anti-GFAP (glial fibrillary acidic protein) antibody (1:1000; Dako, Hamburg, Germany), and axonal injury was investigated with a monoclonal mouse antibody against APP (amyloid beta precursor protein) (1:2000; Chemicon) as described previously by Tauber et al. [27]. For staining of young post-mitotic neurons an antibody directed against calretinin was used (1:1000, Swant, Bellinzona, Switzerland) [28]. For detection of *S. suis* antigen brain sections were blocked with 2% bovine serum albumin (BSA) plus 0.2% Triton X-100 in PBS for 20 min and then incubated with a rabbit anti-*S. suis* antibody (1:250). Secondary biotinylated sheep anti-mouse antibodies (1:200; Amersham, Buckinghamshire, UK) or biotinylated donkey anti-rabbit antibodies (1200; GE Healthcare, Buckinghamshire, UK) diluted in PBS, followed by addition of avidin−biotin peroxidase complex (Vector Laboratories, Burlingame, CA), and diaminobenzidine (Roche, Mannheim, Germany) as chromogenic substrates were used for visualization. Binding of the APP antibody was visualized using the alkaline phosphatase/anti-alkaline phosphatase method with New Fuchsin as the chromogenic substrate. Brain sections were counterstained with hemalum (Merck, Darmstadt, Germany). Isotypic antibodies and brain sections incubated with secondary antibodies in the absence of primary antibodies served as controls as described by Tauber et al. [27]. After incubation with primary and secondary antibodies samples were washed with PBS.

Apoptotic neurons in the dentate gyrus of the hippocampal formation [7, 8, 20, 27] and granulocytes in meningeal infiltrates [29, 30] were identified by in-situ tailing (IST) and morphology (condensed, shrunken nuclei, condensed and shrunken eosinophilic cytoplasm) [31]. The sections were counterstained with nuclear fast red-aluminiumhydroxide (Roche, Mannheim, Germany).

**Fig. 1:**
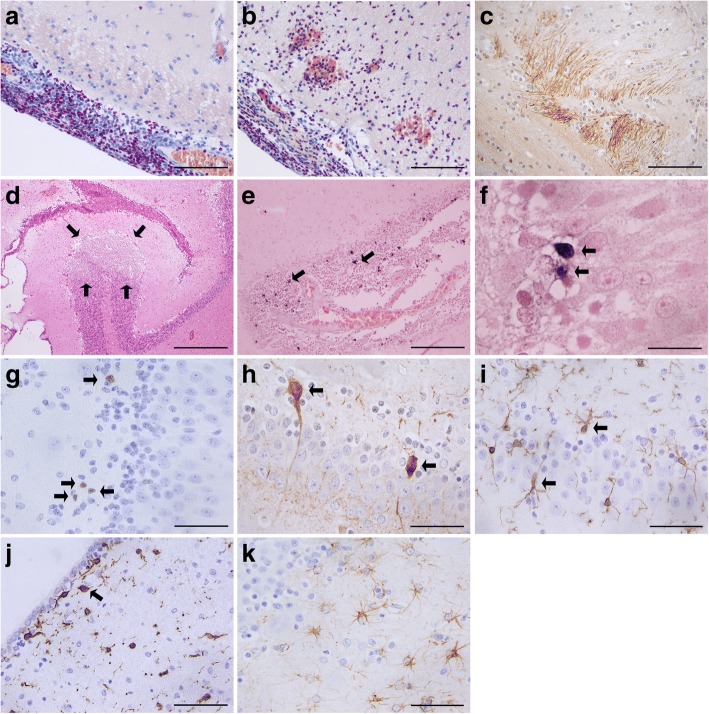
Histology, immunohistochemistry and in-situ tailing of brain sections from piglets suffering from *S. suis* meningitis. Chloroacetate esterase (CAE)-staining of a frontal cortex section of a piglet suffering from meningitis (**a**; animal no. 10) or haemorrhagic meningoencephalitis (**b**; animal no. 3). Neutrophilic granulocytes and some monocytes are stained violet. Axonal damage was visualized by an anti-amyloid beta precursor protein (APP) antibody (brown) (**c**; animal no. 3) and was observed only in one piglet. Ischaemic injuries were visualized by H&E staining and also found only in one animal (**d**; animal no. 3). The densities of apoptotic leukocytes in meningeal infiltrates (**e**; animal no. 4) and granule cells in the hippocampal dentate gyrus (**f**; animal no. 5) were assessed by in-situ tailing (dark violet) and morphology. Proliferation of neural progenitor cells in the dentate gyrus was detected by staining of the proliferating cell nuclear antigen (PCNA) (brown) (**g**; animal no. 4). Young neurons were stained with an anti-calretinin antibody (brown) (**h**; animal no. 5). Microglia were detected by an anti-ionized calcium binding adaptor molecule 1 (Iba-1) antibody (brown) (animal no. 9). Microglia were slightly activated in the dentate gyrus (**i**) and highly activated (**j**) in close proximity to the CSF compartments. Astrocytes were stained with an anti-glial fibrillary acidic protein (GFAP) antibody (brown) (**k**; animal no. 4). The horizontal bars indicate 100 μm (**a**-**c**), 500 μm (**d**), 200 μm (**e**), 20 μm (**f**) and 50 μm (**g**-**k**)

Representative results of stained brain sections are shown in Fig. 1.

### Microscopy and statistical analysis

To quantify cells in the dentate gyrus, only immunoreactive cells in the granule cell layer and the subgranular zone (SGZ) were counted [27]. Analysis Software Imaging System (microscope BX51; Olympus; software AnalySIS 3.2; Soft Imaging System GmbH, Münster, Germany) was used to measure the area of the dentate granule cell layer. For analysis of microglial activation morphology, we followed the activation steps identified and described by Kreutzberg [32] (0 = fully resting microglia with multiple branches; 1 = mix between resting and mildly activated microglia, demonstrating shortened processes with increased thickness; 2 = mildly activated microglia with shortened processes with increased thickness; 3 = mix between mildly and strongly activated ameboid microglia without processes; 4 = ameboid microglia without processes) in the neocortical layers I-III.

Data were expressed as medians and scatter dot plots and compared by two-tailed Mann-Whitney U-test. Statistical analyses were carried out using GraphPad Prism (GraphPad 6 Software, San Diego, CA, USA). Probabilities lower than 0.05 were considered statistically significant (* *p* < 0.05; *** *p* < 0.001).

## Results

In this study the brains of piglets which had been infected intranasally with *S. suis* serotype 2 strain 10 were analysed for leukocyte infiltration, microglial and astrocyte density, ischemia, axonal injury, neuronal apoptosis and neural proliferation in the dentate gyrus of the hippocampal formation as well as microglial activation in the neocortical layers I-III. At autopsy, piglets showed severe diffuse purulent meningitis (5 cases) (Fig. 1a), severe diffuse purulent meningitis with encephalitic involvement of the brain tissue (2 cases) (Fig. 1b), severe multi-focal purulent meningitis (1 case) and mild focal purulent meningitis (2 cases). Axonal damage and ischaemic injury was found in one case (Fig. 1c and d, respectively). Piglets that developed meningitis showed typical signs of illness starting from day 1 until 6.5 days post infection (median time 2 days). Depending on the severity of clinical signs, the piglets were euthanized 0.5–2 days (median time 1.25 days) after onset of the first clinical signs of illness. In the meningitis group *S. suis* was detected by bacteriological cultures and PCR analysis in 8 out of 10 CSF samples and in all brain swabs (Table 1). The control group consisted of 14 piglets that had been intranasally infected but which lacked any *S. suis*-associated lesions in the brain and were negative for the infection strain in the bacteriological screenings (with exception of the tonsils).

**Fig. 2:**
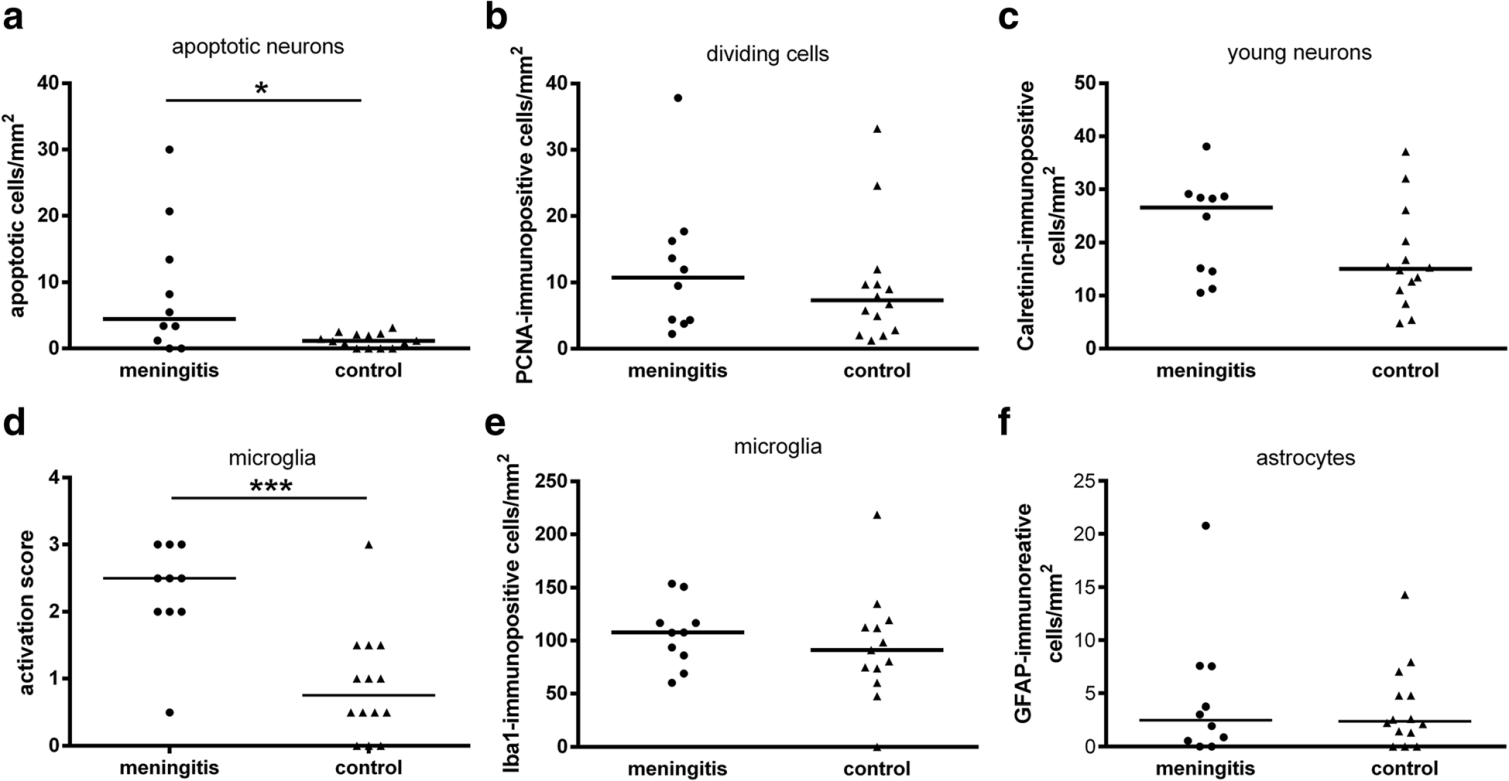
Comparison of densities of immunoreactive cells and morphological scoring of microglia in 10 piglets suffering from meningitis and 14 control piglets. In sections of the hippocampal dentate gyrus, significantly more apoptotic neurons were found in piglets with meningitis compared to control piglets (in-situ tailing) (**a**). No significantly different densities of dividing cells (proliferating cell nuclear antigen (PCNA) staining) (**b**), young neurons (calretinin staining) (**c**), microglia (ionized calcium binding adaptor molecule 1 (Iba-1) staining) (**e**) and astrocytes (glial fibrillary acidic protein (GFAP) staining) were detected (**f**). Morphological scoring of Iba-1 stained cells in the neocortex revealed a significantly stronger activation of microglia in piglets with meningitis (**d**). *p ≤ 0.05; ***p ≤ 0.001

**Fig. 3:**
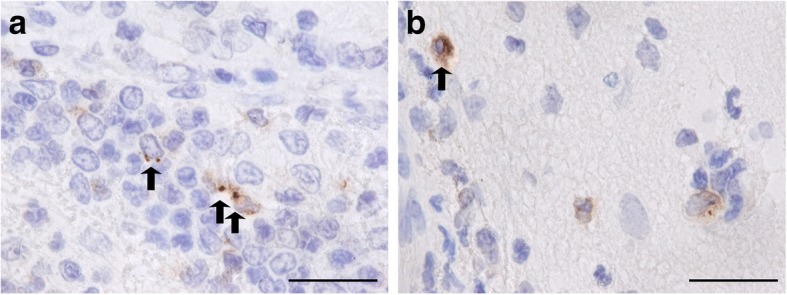
Detection of *S. suis and S. suis* antigens in brain sections of piglets with meningitis. *S. suis* and *S. suis* antigens (brown) were visualized by a *S. suis*-specific antibody in meningeal infiltrates as indicated by arrows (animal no. 5) (**a**) and in the cytoplasm of phagocytes (animal no.2) (**b**). The horizontal bars indicate 20 μm

The hippocampal formations of the piglets were analysed by immunohistochemistry and in-situ tailing for morphological alterations. An increased density of apoptotic neurons in the hippocampal dentate gyrus (median = 4.45/mm^2^) in piglets with meningitis was measured compared to control piglets without signs of CNS inflammation (median = 1.14/mm^2^) (*p* = 0.017) (Fig. 2a). Proliferation in the granule cell layer and the subgranular zone of the hippocampal dentate gyrus as a sign of regeneration, analysed by counting cells positive for PCNA, was slightly increased in the meningitis group but did not differ significantly from the control group (*p* = 0.4) (Fig. 2b). A tendency towards an increased neurogenesis during meningitis was detectable as assessed by the density of calretinin-positive cells, but the difference also did not reach statistical significance (*p* = 0.19) (Fig. 2c). Scoring of the morphology of microglia revealed a strong activation of these cells in the meningitis group and only a slight activation in the control group in layer I-III in the neocortex (Fig. 2d) (*p* = 0.0008). In line with these results, strong microglial activation was seen close to the CSF compartments in piglets suffering from meningitis (Fig. 1j). However, the number of microglial cells did not differ significantly in both groups (Fig. 2e) (*p* = 0.44). We did not see astrocytosis in the hippocampal dentate gyrus (*p* = 0.99, piglets with meningitis versus control piglets) (Fig. 2f). In the meningitis group, *S. suis* and *S. suis* antigens were detected by a *S. suis*-specific antibody in meningeal infiltrates (Fig. 3a) and in the cytoplasm of phagocytes, respectively (Fig. 3b).

## Discussion

In the present study we aimed at assessing whether the morphological alterations seen in the brains of piglets with *S. suis* meningitis are comparable to the abnormalities observed in human autopsy cases and in rodent models of bacterial meningitis. All piglets analysed in this study were infected intranasally with *S. suis*. Piglets which were selected as controls did not show infection-associated lesions of the CNS when investigated histopathologically and were negative in the bacteriological screening for the infection strain in CSF as well as in punctures of the tarsal joints, brain, peritoneal, pleural, and pericardial cavities, cranial lobe of the lung, liver and spleen. Animals were excluded from the analysis as they showed histopathological alterations in more than one organ other than the brain and/or because bacteria were detected in the respective organ(s). All piglets used in the experiments were of similar age and the same breed, but from different litters. It is known that a substantial within-breed variability exists in pigs [33] which may explain the different disease outcomes. Moreover, the course of the disease depends on the immune status of the animals, which is shaped by genetics, transferred maternal immunity (at least in the first weeks after birth), health status and environmental factors [34, 35]*.*

Meningitis is a typical feature of *S. suis* CNS infections, whereas encephalitis (i.e., involvement of brain tissue) is described less frequently. In the present study, we observed meningitis in 8 out of 10 piglets with infection of the CNS and in two out of 10 piglets a strong meningoencephalitis. Similarly, in humans infected with *S. suis*, the main pathology is meningitis followed by sepsis, arthritis, endocarditis, and endophthalmitis [5]. Encephalitic involvement of the brain parenchyma was described less frequently in patients [36, 37]. In an experimental model using the Chinese *S. suis* serotype 2 strain SC19, intranasal inoculation of pigs resulted in meningoencephalitis in all animals with CNS infection [38]. Similarly, Sanford [39] reported meningoencephalitis and meningoencephalomyelitis in all 53 investigated cases of naturally occurring *S. suis* CNS infections in pigs. In contrast, others found only meningitis after experimental infection [40–43]. Involvement of the brain tissue may depend on serotype [44], strain and (in experimental infections) on study design*.*

Inflammation of the CNS was observed in 10 out of 35 piglets (28.6%) after intranasal inoculation of *S. suis*. In a murine colonization and infection model, meningitis was detected only in 1 out of 10 mice (without intranasal pretreatment with acetic acid) and in none of 32 mice in which the colonization resistance of the nasal mucosa was reduced by intranasal administration of 1% acetic acid, even though the same *S. suis* strain and a five times higher infection dose (5 × 10^9^ CFU/mouse) was used [45]. In a murine pneumococcal infection model meningitis rates of 50% were observed after intranasal administration of hyaluronidase prior infection [10].

In most animal models, and in human autopsy cases, neuronal injury and neural repair as well as microglial proliferation and astrocytosis are frequently detectable in the hippocampal formation during meningitis and meningoencephalitis [7, 8, 17, 20, 27, 46–50]. Therefore, we focused on morphological changes in this brain region during CNS infection with *S. suis*. In piglets suffering from meningitis, we observed an increase in apoptotic neurons in the dentate gyrus compared to control piglets. These results are in line with morphological abnormalities in a rabbit model of pneumococcal meningitis [8, 47], in neonatal and adult rat models [7, 51] and in human autopsy cases [20]. However, the number of apoptotic cells was in general lower in the pig model (meningitis group: 0–30 apoptotic cells/mm^2^ vs. control group: 0–3 apoptotic cells/mm^2^) in contrast to the rabbit model (meningitis group: 227 (99/339) apoptotic cells/mm^2^ vs. control group: 41 (37/45) apoptotic cells/mm^2^, [median (25./75. quartile)]) [47] but comparable with a mouse model of pneumococcal meningitis (meningitis group: 71 ± 29 apoptotic cells/mm^2^ vs. control group: 37 ± 18 cells/mm^2^) [52]. The density of apoptotic dentate granular cells in our piglet model was similar to the density of neuronal apoptosis in human autopsy cases after death from meningitis [20]. Cytotoxic activity of the secreted cytolysin suilysin of *S. suis* may directly induce neuronal injury as demonstrated by Stringaris et al. for pneumolysin [53], the highly homologous protein of *S. pneumoniae* [54]. A further well described mechanism involved in neuronal damage is the activation of microglial cells. Different stimuli such as CpG-DNA [55]; LPS [56, 57], Pam_3_CSK_4_ [58] or beta-Amyloid [59, 60] were shown to activate microglia, causing neuronal damage. In addition, the gram-positive cell wall can activate microglia [61]. Scoring of the microglial morphology revealed a statistically significant activation of microglia in the meningitis group (Fig. 2d). However, we detected only a slight increase in microglial cell numbers in piglets with meningitis even though increased inflammation causes microglial proliferation in the human CNS [27, 48].

In the present study, we observed a tendency towards increased proliferation and neuronal differentiation during *S. suis* CNS infection as signs of regeneration. In humans, significant proliferation of progenitor cells in the hippocampal dentate granule cell layer and subgranular zone was observed during bacterial meningitis [17], septic metastatic encephalitis [49] and fungal meningoencephalitis [48]. Different animal models of pneumococcal CNS infection revealed similar results: neurogenesis peaked at day 2 post infection, and the difference between infected and uninfected mice reached statistical significance at day 6 after intracerebral infection (measured by density of BrdU-labeled cells in the subgranular layer of the dentate gyrus). Likewise, neurogenesis was significantly increased in rabbits 24 h after intracisternal infection with pneumococci [47] and in rats 7 days post infection [62]. In contrast to the mouse, rat and rabbit models [7, 47, 62–64], piglets were infected by intranasal inoculation which reflects the natural route of infection and were not treated with antibiotics.

The lack of significant changes in regard to neuronal proliferation and differentiation as well as astrocytosis and number of microglia, which are observed in human autopsy cases [17, 27, 48], is most likely the result of the short period of time between the onset of clinical symptoms such as fever or apathy and organ sampling. In line with this hypothesis, axonal damage and ischaemic injury were only observed in one piglet, even though microbial compounds can cause axonal damage via stimulation of immune cells [65] and ischaemic injuries are observed in cases of human meningitis [21]. Piglets were killed as early as 12–48 h after onset of the first clinical signs (median interval 30 h) due to reasons of animal welfare. An antibiotic treatment 12 or 24 h post infection [20, 47] would be prerequisite for extending the survival time of piglets, and thus increase the likelihood of detecting more marked morphological changes.

Unlike in rodents, several sensitive and valid test batteries are available for the assessment of long-term motor and neuropsychologic sequelae in pigs [14, 15]. In addition, the pig is considered to be an excellent model for studying infectious diseases of humans [13, 66]. Genetics, anatomy and physiology of pigs are closely related to humans. In regard to immunology, the porcine immune system closely resembles the human immune system for more than 80% of analyzed immunological parameters, in stark contrast to mice in which less than 10% of the parameters analyzed were more similar to humans (see review by Meurens et al. [13]).

## Conclusions

The pig model of *S. suis* meningitis by intranasal infection appears to be most suitable for studying treatment options of bacterial meningitis. It has the advantage that it more closely resembles human meningitis than rodent models, as underlined by our findings that the brains of piglets showed the typical morphological alterations present in human autopsy cases. The lack of statistically significant differences of some parameters in the present study may be the consequence of the short duration of infection and of a mild inflammatory response in the control group due to the intranasal inoculation of bacteria. As shown in rodent infection models [29, 62, 67, 68] an antibiotic treatment can prolong survival and increase the chance of detecting stronger morphological alterations caused by infectious pathogens. Therefore, the pig should be considered as a model for evaluating the effects of experimental therapeutic approaches during CNS infections.

## Abbreviations

APP: Amyloid beta precursor protein
BSA: Bovine serum albumin
CAE: Chloroacetate esterase
CFU: Colony forming units
DPI: Days post infection
CNS: Central nervous system
CSF: Cerebrospinal fluid
epf: *Extracellular factor*
FCS: Fetal calf serum
GFAP: Glial fibrillary acidic protein
h: Hours
HE: Haematoxylin and eosin
Iba-1: Ionized calcium binding adaptor molecule-1
IST: In-situ tailing
mrp: *Muramidase-released protein*
PBS: Phosphate-buffered saline
PCNA: Proliferating cell nuclear antigen
S: *Streptococcus*
SGZ: Subgranular zone
sly: *Suilysin*
